# Indoor air pollution and its association with poor lung function, microalbuminuria and variations in blood pressure among kitchen workers in India: a cross-sectional study

**DOI:** 10.1186/s12940-017-0243-3

**Published:** 2017-04-04

**Authors:** Amarnath Singh, Chandrasekharan Nair Kesavachandran, Ritul Kamal, Vipin Bihari, Afzal Ansari, Parappurath Abdul Azeez, Prem Narain Saxena, Anil Kumar KS, Altaf Hussain Khan

**Affiliations:** 1 Epidemiology Laboratory, Systems Toxicology and Health Risk Assessment Group, CSIR-Indian Institute of Toxicology Research (CSIR-IITR) , Vishvigyan Bhavan, 31, Mahatma Gandhi Marg, Lucknow, 226001 Uttar Pradesh India; 2 Advance Imaging Facility, CSIR-Indian Institute of Toxicology Research (CSIR-IITR), Vishvigyan Bhavan, 31, Mahatma Gandhi Marg, Lucknow, 226001 Uttar Pradesh India; 3 Salim Ali Centre for Ornithology and Natural History, Ministry of Environment, Forest and Climate Change, Government of India, Anaikatty, Coimbatore, 641108 Tamil Nadu India; 4 Medicinal and Process Chemistry Division, CSIR-Central Drug Research Institute (CSIR-CDRI), Sector-10, Jankipuram Extension, Sitapur Road, Lucknow, 226031 Uttar Pradesh India; 5 Environmental Monitoring Laboratory, Environmental Toxicology Group, CSIR-Indian Institute of Toxicology Research (CSIR-IITR), Vishvigyan Bhavan, 31, Mahatma Gandhi Marg, Lucknow, 226001 Uttar Pradesh India; 6 Department of Biochemistry, Babu Banarasi Das University, BBD City, Faizabad Road, Lucknow, 226028 Uttar Pradesh India

**Keywords:** Indoor air, Microalbuminuria, Lung function, Blood pressure, Kitchen workers

## Abstract

**Background:**

The present study is an attempt to explore the association between kitchen indoor air pollutants and physiological profiles in kitchen workers with microalbuminuria (MAU) in north India (Lucknow) and south India (Coimbatore).

**Methods:**

The subjects comprised 145 control subjects, 233 kitchen workers from north India and 186 kitchen workers from south India. Information related to the personal and occupational history and health of the subjects at both locations were collected using a custom-made questionnaire. Worker lung function was measured using a spirometer. Blood pressure was monitored using a sphygmomanometer. Urinary MAU was measured using a urine analyzer. Indoor air monitoring in kitchens for particulate matter (PM), total volatile organic compounds (TVOC), carbon dioxide (CO_2_) and carbon monoxide (CO) was conducted using indoor air quality monitors. The size and shape of PM in indoor air was assessed using a scanning electron microscope (SEM). Fourier transform infrared (FTIR) spectroscopy was used to detect organic or inorganic compounds in the air samples.

**Results:**

Particulate matter concentrations (PM_2.5_ and PM_1_) were significantly higher in both north and south Indian kitchens than in non-kitchen areas. The concentrations of TVOC, CO and CO_2_ were higher in the kitchens of north and south India than in the control locations (non-kitchen areas). Coarse, fine and ultrafine particles and several elements were also detected in kitchens in both locations by SEM and elemental analysis. The FTIR spectra of kitchen indoor air at both locations show the presence of organic chemicals. Significant declines in systolic blood pressure and lung function were observed in the kitchen workers with MAU at both locations compared to those of the control subjects. A higher prevalence of obstruction cases with MAU was observed among the workers in the southern region than in the controls (*p* < 0.01).

**Conclusions:**

Kitchen workers in south India have lower lung capacities and a greater risk of obstructive and restrictive abnormalities than their north Indian counterparts. The study showed that occupational exposure to multiple kitchen indoor air pollutants (ultrafine particles, PM_2.5_, PM_1_, TVOC, CO, CO_2_) and FTIR-derived compounds can be associated with a decline in lung function (restrictive and obstructive patterns) in kitchen workers with microalbuminuria. Further studies in different geographical locations in India among kitchen workers on a wider scale are required to validate the present findings.

## Background

Cooking oil fumes at workplaces such as kitchens contain polycyclic aromatic hydrocarbons (PAHs), volatile organic compounds, aldehydes, alkanoic acids, carbon monoxide, carbon dioxide, and fine and ultrafine particulate matter and can be a major source of indoor air pollution [1, 2]. These pollutants have been associated with increased cardiovascular mortality and morbidity [3, 4]. According to the 2014 World Health Organization (WHO) report, particulate matter (PM) is responsible for approximately 7 million deaths worldwide [5]. India has the highest mortality rate due to indoor and outdoor air pollution sources [6]. Indoor cooking at high temperatures (>300 °C) in a kitchen can generate different types of PM and toxicants including aldehydes, PAHs, heterocyclic amines, aromatic amines, and alkanoic acids [2, 7]. These indoor aerosols also contains fatty acids, short-chain aldehydes, higher aldehydes, and fine and ultrafine particles (UFP) and pose risks for cooks in commercial kitchens [2]. PM can be classified according to its aerodynamic diameter into size fractions such as PM_10_ (“thoracic” particles, < 10 μm), PM_2.5–10_ (“coarse” particles, 2.5 to 10 μm), PM_2.5_ (“fine” particles, < 2.5 μm) and UFP (<0.1 μm) [8]. The particles with very small size and high surface-area-to-mass ratio carry several toxicants and are deposited into human lung alveoli, where they activate multiple pathophysiological processes [9] as well as inflammation and oxidative stress [10]. Both acute and chronic exposure to fine and ultrafine particle have been associated with heart failure [11], cerebrovascular disease [12], hypertension [13] and lung function abnormalities of the obstructive and restrictive types [14, 15]. In vitro and in vivo studies have demonstrated that UFP can pass directly into the circulation and induce inflammation [16].

Inflammatory markers induced by PM in the lung can reach the systemic circulation and cause indirect endothelial vascular damage [17]. Endothelial dysfunction, transvascular blood albumin leakage and chronic inflammation due to PM exposure can be considered possible causes of cardiovascular problems [18, 19]. Microalbuminuria (MAU) occurs due to leakage of blood albumin from kidneys [20]. The urinary albumin-to-creatinine ratio (ACR) is considered the *‘gold standard’* for the determination of MAU [21, 22]. Thus, MAU is considered an independent primary surrogate marker of kidney disease in patients with hypertension and a strong risk predictor of cardiovascular problems [23, 24] including atherosclerosis [25]. Previously, MAU was considered a causative factor for nephropathy in patients with diabetes [26], but several subsequent studies have reported that MAU is a major cause of mortality even in individuals without diabetes [27, 28]. In a Korean population, the relationship between MAU and poor lung function was reported as an early surrogate marker of kidney damage and cardiovascular problems [20]. An inverse relationship between lung function abnormalities of the obstructive and restrictive types and ACR after adjusting for age, obesity and smoking was also reported in a study in the general population in Korea [20].

Exposure to PM from environmental sources and its relation with morbidity and mortality due to cardiorespiratory problems are well known. MAU caused by environmental exposure to PM is not well understood, except for a report on the incidence of MAU among local residents after the 9/11 World Trade Center attacks in the United States due to PM exposure [29]. Occupational exposure to PM and its association with MAU were not addressed in any other previous studies. The present cross-sectional study is an attempt to explore the association of lung function abnormalities and blood pressure variations in kitchen workers with MAU exposed to PM and other kitchen indoor air toxicants at two regional locations in India. The study will help policy holders implement measures to minimize indoor air pollution in commercial kitchens.

## Methods

### Study design and subjects

A cross-sectional study was conducted among male kitchen workers in north India (Lucknow) and south India (Coimbatore). The inclusion criteria for the kitchen workers were age 18–60 years, job experience for at least the past three years, and absence of any communicable diseases during the study period. Furthermore, kitchen workers who were smokers or who consumed alcohol, caffeine, vitamin supplements or medicines such as psychotropic drugs, antihypertensive drugs and antihistamines were excluded from the study as those substances interfere with the parameters monitored during the study. The inclusion criteria for the control subjects were age 18–60 years, employment in a non-kitchen area for at least the past three years in the north or south of India (housekeeping and office staff), similar socio-economic status to kitchen workers and absence of any communicable diseases during the study period. The exclusion criteria used in the case of kitchen workers were also applicable for control subjects. Both kitchen workers and control group subjects worked from 09.00 to 16.00 h, with a minimum of seven hours a day. In total, 564 workers who satisfied the inclusion criteria were examined in the study. Of these, 145 were non-kitchen workers (controls); 233 kitchen workers from north India and 186 kitchen workers from south India were pooled for the analysis. Those workers who were absent from duty on the dates of the survey were also excluded.

A questionnaire was prepared by the authors and amended based on the workers’ reactions to the questions following a pretest in the field. Personal details, health and occupational history of each of the subjects were recorded in the pretested questionnaire.

### Ethics, consent and permission

Ethical clearance for the study was obtained from the Institutional Human Ethics Committee, CSIR-Indian Institute of Toxicology Research, Govt. of India, Lucknow. Only participants who gave voluntary written informed consent to participate in the study were included in the survey.

### Clinical physiological measurements

Postprandial blood glucose testing was conducted 2 h after the workers’ routine breakfast (carbohydrate diet). The pad of their finger was wiped with alcohol, allowed to dry and then punctured with a sterile lancet (Roche Diagnostics, Germany), and blood was drawn onto the test strip, preloaded in the glucometer. The glucometer (Accu-Chek Active, Roche Diagnostics, Germany) reports blood glucose measurement in mg/dL within 15–30 s. If the blood sample was inadequate, the test was repeated using a new strip. The recommended guideline on self-monitoring of blood glucose as per International Diabetes Federation (IDF) was followed in the study. The normal postprandial glucose level was considered to be <140 mg/dL as per the IDF. Systolic blood pressure (SBP), diastolic blood pressure (DBP) and pulse were measured by standard methods using a sphygmomanometer with the patient in a sitting position. Normal blood pressure was defined as SBP below 120 mmHg and DBP below 80 mmHg [20]. Body mass index (BMI) was calculated using the standard method.

### Lung function test

Peak expiratory flow rate (PEFR), forced vital capacity (FVC), forced expiratory volume in one second (FEV_1_) and forced expiratory volume in six seconds (FEV_6_) were measured for the study subjects using a spirometer (PIKO-1 and PIKO-6, Ferraris Cardiorespiratory, Louisville, USA) following the specifications of the American Thoracic Society (ATS). The highest values of PEFR, FEV_1_ and FEV_6_ of three tests on each subject were recorded. The procedure followed for the test followed the recommendations of the ATS [30]. The obstructive airflow pattern was defined as FEV_1_/FEV_6_ ratio < 0.7 [20]. Restriction was classified using the GOLD criteria of FVC < 80% [31]. Predicted values for FVC or FEV_6_, FEV_1_ and PEFR were calculated using Indian norms [32].

### Urine analysis for microalbuminuria (MAU)

Each individual subject submitted a fresh, random, mid-stream urine sample for the analysis, which was collected in a sterile container early in the morning and stored at −80 °C for further analysis. Dipstick-based urinalysis was conducted using Clinitek Microalbumin 2 strips (Siemens Healthcare Diagnostics ltd, Frimley, UK) for urine albumin and creatinine determination, from which urinary ACR was estimated for MAU with a U-Check Analytics sensor (Biosense Technologies Pvt. Ltd, Mumbai, India). MAU is defined as urinary ACR (30–300 mg/24 h) [33]. The subjects were characterized based on ACR ratio (30–300 mg/g) into two groups, viz., MAU and non-MAU.

### Air quality studies- Sampling site and hygiene practices

Indoor air quality in kitchen air can be influenced by different cooking methods, the fuels used for cooking and ventilation conditions. To understand indoor air pollutants generated in cooking practices, a survey of indoor air quality monitoring was conducted in each kitchen in the peak hours of food preparation (9.00-16.00 h) in both locations. The kitchens studied in northern and southern India prepared food for more than 3000 people daily for breakfast, lunch, evening snacks and dinner. Both kitchens were using liquefied petroleum gas (LPG) as fuel for the gas stoves with efficient range hoods. The range-hood chimneys used in the kitchen were of “low sidewall” type. Each kitchen was 1500–2000 square feet in area and had 2–3 hood ventilators, 4–6 exhaust fans and fresh air blowers. During air sampling in the kitchens, it was ensured that the doors of the restaurants and windows of the kitchens were kept closed in order to create a pure indoor environment and exclude pollution from outside. The ventilation hoods to expel the cooking fumes were kept open during indoor air monitoring. The kitchen floor was regularly cleaned with germicidal solutions. The worktables of the kitchen were cleaned after each dish preparation. The kitchen workers wore aprons and head coverings while in the kitchen. They also washed their hands with antiseptic soap solution before and after each cooking activity. Dishes were cleaned in a separate room away from the kitchen premises. Irrespective of their locations in north and south India, the oils used were refined vegetable oils for vegetarian and non-vegetarian dishes. The indoor air quality parameters recorded in different kitchens at both locations were pooled for the analysis.

### Indoor air monitoring of pollutants

In most studies, the air samplers were placed on the exit of the exhaust duct at the roof of the restaurants or hotels, which disperse the air pollutants from the kitchen to the ambient air. Possibly for that reason, such studies failed to correlate their indoor air observations with the respiratory health of the workers. In the present study, the air samplers were located 0.5 m from the cooking pan and 1.5 m above the ground to simulate the breathing zone [34]. Indoor air monitoring in the breathing zone of the kitchen worker, as in the present study, is the appropriate monitoring strategy for health risk assessment among kitchen workers. Sampling was performed at a single site in the kitchen where workers were engaged in cooking. The refined oils, a major ingredient, used for cooking in the two kitchens were similar. In north Indian cooking, wheat flour is the base ingredient, while in south India, it is rice. Frying is done regularly in both the kitchens, in the north and in the south, for snacks and breakfast items. Meat preparations are either fried or prepared with gravy at both the locations.

The particulate matter concentrations (PM_1_ and PM_2.5_) in indoor air of kitchens were monitored using portable particulate matter measuring equipment (HAZ-DUST, Model EPAM-5000, Environmental Devices Corporation, New Hampshire, USA). Particulate matter was also collected on a glass microfiber filter in the sampling head connected with a silicon tube using a Millipore pump (General Electric, Fort Wayne, Indiana) with a flow rate of 1.5 L/min. The sampling devices were protected against light during and after sampling by wrapping in aluminum foil. The PM samples (on the filter paper) were used for particle size characterization and elemental composition using scanning electron microscope and energy-dispersive X-ray (SEM-EDX) analysis. CO and CO_2_ in the kitchen were monitored using indoor air quality monitors (IQM 60, Aeroqual, New Zealand). The concentration of total volatile organic compounds (TVOC) in the kitchen was monitored using a portable handheld VOC monitor (MiniRAE 3000, RAE Systems, USA). Using the same sampling devices, control air samples were also collected from non-kitchen space (non-tobacco smoke zones) to determine indoor air pollutants present in the breathing zone of the control subjects.

### Scanning electron microscope and energy-dispersive X-ray (SEM-EDX) analysis of kitchen indoor air samples

Particulate matter was examined for the shape, size, surface morphology and elemental composition of individual particles by high-resolution SEM (Quanta FEG450, FEI, Netherland). From the PM sample (dry filter paper), 1 mm^2^ from the center of each filter paper was taken for SEM analysis. The particulate matter samples were placed on double-stick conducting carbon tape over an aluminum stub and coated with gold under an argon atmosphere by means of a sputter coater (SC7620, Mini sputter coater, Quorum Technology Ltd., U.K.). The fine coating of gold, applied using vacuum coating unit, makes the aerosol sample more conductive. The shape, size, and surface morphology of particulate matter was analyzed by a secondary electron detector at an acceleration voltage of 5.0-10.0 kV at a working distance of 9–10 mm. Images of each sample were taken thrice at different magnification ranging from x5000 to x80000.

The elemental composition of the PM was determined by an EDX detector (APOLLO XL, USA) attached to the SEM. The EDX spectra of individual kitchen indoor air filter paper samples were recorded after they were scanned with an electron beam with a scan speed of 300 ns/pixel at an acceleration voltage of 20 kV. The EDX system has a detection limit of >1% and an energy resolution of 130 eV. The elements present were measured both qualitatively and quantitatively. The EDX spectra of a blank filter were also obtained, and its elemental composition was manually subtracted during the evaluation of EDX spectra of kitchen indoor air samples.

### Fourier transform infrared spectroscopy analysis of indoor air kitchen samples

Aerosols collected on glass microfiber filters were analyzed for spectra by FTIR spectroscopy. All filter paper samples were equilibrated and weighed as per the EPA protocol [35] before and after the sampling procedure. A total of 9 samples, including blanks, were analyzed using an FTIR spectrometer (Perkin Elmer 881 model, wavenumber range 450–4000 cm^−1^, 40 scans/sample and 1 cm cell path length). The trapped indoor air toxicants were extracted from the filters with 5 ml of a 1:1 *v/v* mixture of dichloromethane (Spectrochem) and absolute ethanol (Merck) and later filtered through a sintered funnel. The filtrate was evaporated under a vacuum until pale yellow sticky oil (trace in quantity) was formed. The analysis of the filtrate was directly determined and recorded using the FTIR spectrometer.

### Statistical analysis

Data from kitchen workers were grouped into workers with microalbuminuria (MAU) and those without microalbuminuria (non-MAU) for comparative analysis. Descriptive statistics (mean, standard deviations and range) were calculated for all quantitative variables. All the categorical variables are presented as frequencies and their percentages. Extreme outliers in the environmental and health data were identified using box plots. These extreme values were then excluded from the statistical analysis, since their presence can lead to inflated error rates and distortion of estimates with either parametric or nonparametric tests. The significance for categorical variables was assessed using a chi-squared test. One-way ANOVA was used to compare the mean values among the quantitative variables, followed by a Bonferroni post hoc test to compare the mean values among the various groups. The concentrations of the indoor air pollutants among the three locations were compared using appropriate statistical tests, viz*.*, one-way ANOVA in the case of normally distributed data and the Kruskal-Wallis test for non-parametric data. The odds ratio and 95% confidence interval (CI) were calculated using multiple logistic regression models for microalbuminuria with respect to pulmonary function in MAU subjects after adjustment for confounders. Univariate logistic regression analysis was carried out to identify the potential confounders, which were then adjusted in the multivariate analysis. The criterion of significance was set at *p* < 0.05. All calculations were performed using STATA software (version IC 13, Stata Corp LP, TX, USA).

## Results

The physical characteristics, personal habits and physiological profiles of the study subjects are given in Table 1. There was no significant difference in height, weight, body mass index, smoking history, or alcohol intake between the comparison groups. However, a statistically significant difference was observed in the mean ages between some groups: non-MAU vs. MAU groups of kitchen workers, and kitchen workers vs. control groups. Therefore, necessary statistical corrections were undertaken to nullify the confounding effect of age on the physiological profiles. High SBP was observed among the kitchen workers with MAU from the north and south region compared to that of their corresponding controls with MAU. No difference in DBP, pulse or postprandial random glucose level was observed among the comparison groups with or without MAU (Table 1).

**Table 1:** Demographic Characteristics, personal habits, physiological profiles of study subject

Parameters	Control		Kitchen workers (North India)		Kitchen workers (South India)	
	Non MAU (*n* = 107)	MAU (*n* = 38)	Non MAU (*n* = 101)	MAU (*n* = 132)	Non MAU (*n* = 108)	MAU (*n* = 78)
Age, Mean ± SD (Range)	31.51 ± 9.50 (18 – 60)	37.0 ± 12.30 (19 – 60)	27.82 ± 7.62 (19 – 51)	29.65 ± 9.45*** (18 – 58)	27.94 ± 10.58 (18 – 60)	30.78 ± 11.38** (18 – 60)
Height (cm) Mean ± SD (Range)	166.34 ± 6.97 (149 – 180)	165.24 ± 7.23 (150 – 180)	167.82 ± 7.24 (150 – 187)	166.14 ± 6.83 (153 – 188)	164.52 ± 6.02 (150 – 180)	167.06 ± 7.23 (150.5 – 185)
Weight (Kg) Mean ± SD (Range)	64.83 ± 12.72 (42.4 – 109.5)	63.09 ± 16.96 (40.8 – 119)	63.16 ± 12.66 (43 – 98.4)	63.68 ± 11.08 (44 – 104)	61.48 ± 11.96 (42.4 – 103)	63.88 ± 14.15 (34.4 – 100)
Body Mass Index (Kg/m^2^) Mean ± SD (Range)	23.33 ± 3.98 (16.4 – 34.6)	22.95 ± 5.12 (16 – 37.6)	22.36 ± 3.93 (15.6 – 35.5)	23.06 ± 3.45 (15.8 – 34.1)	22.65 ± 4.32 (10.9 – 36.9)	22.83 ± 4.86 (11.4 – 39.8)
Smoking history n (%)	15 (14.02)	6 (15.78)	18 (17.82)	32 (24.24)	24 (22.86)	10 (12.82)
Alcohol consumption n (%)	24 (22.43)	14 (36.84)	17 (16.83)	18 (13.64)	57 (52.78)	37 (47.44)
Systolic Blood Pressure (mmHg) Mean ± SD (Range)	123.35 ± 12.24 (100 – 154)	120.64 ± 10.81 (100 – 150)	123.43 ± 13.71 (100 – 160)	125.61 ± 12.49* (90 – 156)	124.92 ± 14.96 (100 – 220)	125.69 ± 14.42* (100 – 160)
Diastolic Blood Pressure (mmHg) Mean ± SD (Range)	81.87 ± 10.85 (60 – 100)	81.18 ± 8.72 (66 – 98)	83.01 ± 10.35 (60 – 112)	84.88 ± 10.45* (60 – 120)	81.46 ± 10.79 (60 – 120)	83.95 ± 10.96 (60 – 116)
Pulse (beats/min) Mean ± SD (Range)	82.78 ± 10.85 (56 – 116)	84.03 ± 13.67 (60 – 114)	85.07 ± 11.47 (58 – 108)	85.17 ± 13.88 (53 – 136)	82.95 ± 12.68 (58 – 111)	82.40 ± 15.45 (38 – 136)
Blood glucose (mg/dL) Mean ± SD (Range)	120.33 ± 60.57 (70 – 553)	122.33 ± 59.12 (68 – 370)	114.83 ± 22.49 (82 – 197)	120.35 ± 45.96 (68 – 536)	117.78 ± 35.52 (74 – 357)	134.91 ± 80.94 (67 – 490)

All comparisons have been done among the MAU subjects in the three groups. * *p* < 0.05, ** *p* < 0.01, *** *p* < 0.001

The details of lung function among kitchen workers with respect to MAU are given in Table 2. FEV_1_, FEV_6_, PEFR and predicted FEV_6_% among kitchen workers with MAU in south India were lower in terms of volumes and flow rates than those of their respective control groups. Obstructive lung function abnormalities were more prevalent (*p* < 0.01) among the workers in south India than among their counterparts in north India. The odds ratios also indicate higher risk among the south Indian kitchen workers than the respective controls and the comparable group in north India (Table 3). Although there was a statistically significant difference in SBP among kitchen workers, no association was observed between SBP and MAU.

**Table 2:** Alteration in Pulmonary function values with respect to microalbuminuria (MAU)

Parameters	Control		Kitchen workers (North India)		Kitchen workers (South India)	
	Non MAU (*n* = 107)	MAU (*n* = 38)	Non MAU (*n* = 101)	MAU (*n* = 132)	Non MAU (*n* = 108)	MAU (*n* = 78)
FEV_1_ (L/s) Mean ± SD (Range)	2.25 ± 0.56 (1.03 – 3.35)	2.01 ± 0.63 (0.67 – 3.51)	1.95 ± 0.57 (0.91 – 3.58)	1.74 ± 0.56* (0.88 – 2.98)	1.60 ± 0.49 (0.49 – 2.65)	1.30 ± 0.49*** (0.28 – 2.69)
FEV_6_ (L/s) Mean ± SD (Range)	2.49 ± 0.61 (1.26 – 3.78)	2.32 ± 0.66 (0.82 – 4.38)	2.24 ± 0.56 (0.96 – 3.65)	2.06 ± 0.51* (0.88 – 3.19)	1.98 ± 0.53 (0.72 – 3.76)	1.78 ± 0.63* (0.36 – 3.6)
PEFR (L/min) Mean ± SD (Range)	435.06 ± 99.97 (162 – 702)	328.31 ± 97.83 (142 – 650)	415.27 ± 111.04 (151 – 657)	382.71 ± 135.66* (144 – 658)	303.18 ± 90.39 (108 – 572)	296.68 ± 102.29*** (71 – 564)
% predicted FEV_1_% Mean ± SD (Range)	76.94 ± 17.28 (35.86 – 114.73)	70.41 ± 19.74 (29.58 – 127.63)	68.95 ± 19.51 (30.67 – 134.38)	63.03 ± 18.20* (29.98 – 109.76)	57.40 ± 18.06 (14.02 – 106.20)	45.60 ± 17.19*** (14.34 – 104.49)
% predicted FEV_6_% Mean ± SD (Range)	68.86 ± 15.67 (33.41 – 112.46)	64.28 ± 17.48 (24.63 – 117.98)	62.24 ± 16.91 (30.22 – 128.61)	57.56 ± 14.01* (29.71 – 95.17)	57.23 ± 15.51 (21.78 – 103.30)	50.40 ± 17.04* (14.18 – 116.88)
Obstruction (FEV_1_/FEV_6_) n (%)	15 (14.02)	5 (13.16)	4 (3.96)	13 (9.85)	26 (24.07)	30 (38.46)**
Restriction n (%)	77 (71.96)	29 (76.32)	47 (46.53)	81 (61.36)	86 (79.63)	68 (87.18)

All comparisons have been done among the MAU subjects in the three groups. * *p* < 0.05, ** *p* < 0.01, *** *p* < 0.001

**Table 3:** Odds ratio (95% CI) for microalbuminuria with respect to pulmonary function in MAU subjects

Pulmonary function pattern	Control	Kitchen workers (North India)	Kitchen workers (South India)
	OR (95% CI)	OR (95% CI)	OR (95% CI)
Obstruction (FEV_1_/FEV_6_)	0.91 (0.39 – 2.08)	1.46 (0.58 – 3.66)	1.59 (0.98 – 2.57)
Restriction	1.39 (0.79 – 2.48)	1.57 (1.06 – 2.32)	1.63 (1.11 – 2.40)

Models adjusted for age, blood pressure (normal/hypertensive), obesity (using body mass index), smoking history, heavy alcohol drinking

The indoor air quality of kitchens at both locations (south and north India) was given in Table 4. The indoor air quality report clearly indicates higher levels of PM_1_, PM_2.5_, TVOC and CO in kitchens of both locations than in the control locations (non-kitchen areas). The levels of indoor air pollutants are higher in the kitchens located in south India than the kitchens in north India, except in the case of TVOC and CO_2_.

**Table 4:** Indoor air pollutants in kitchen

Parameter	Control location (Non kitchen areas)	North Indian Kitchen	South Indian Kitchen
PM_2.5_ (μg/m^3^) Mean (Range)	34.67 (14 – 57)	68.18 (1–293) ***	78.55 (10 – 429) ***
PM_1_ (μg/m^3^) Mean (Range)	26.75 (10 – 48)	59.46 (1–187) ***	61.52 (8 – 409) ***
TVOC (ppb) Mean (Range)	319.27 (209 – 534)	961.81 (772 – 3124) ***	399.39 (224 – 525)
CO (ppm) Mean (Range)	0.62 (0.6 – 0.7)	6.54 (0.1 – 35.3) ***	11.39 (0.1 – 19.5) ***
CO_2_ (ppm) Mean (Range)	621.3 (611 – 627)	941.66 (579 – 1582) **	661.06 (432 – 1087)

** *p* < 0.01, *** *p* < 0.001

**Fig. 1:**
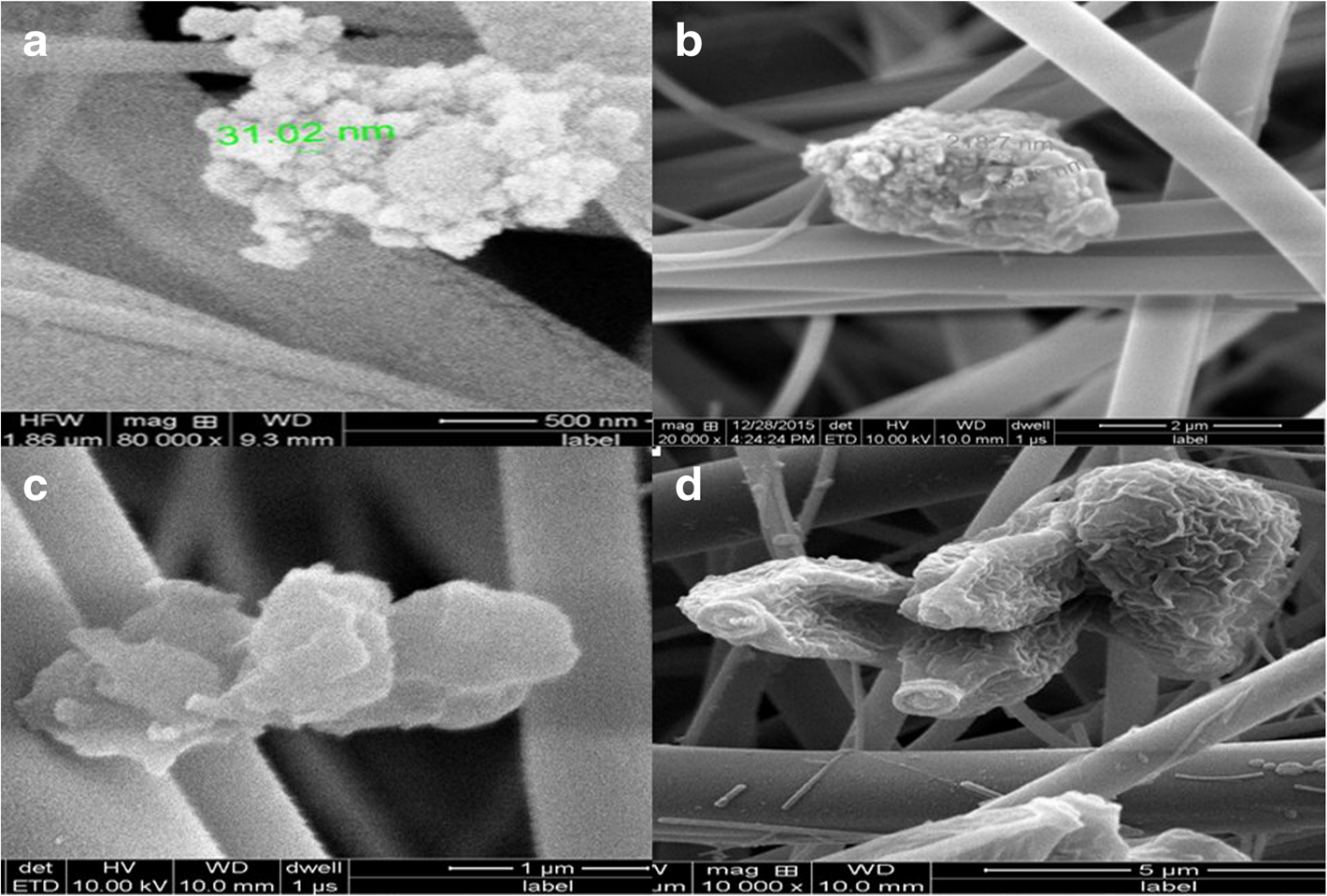
Scanning electron micrograph (SEM) of different shape and size of particulate matter in kitchen indoor air. Scanning electron micrograph of different location kitchen indoor air sample: (1) North Indian Kitchen (**a**,**b**) (2) South Indian Kitchen (**c**,**d**)

**Fig. 2:**
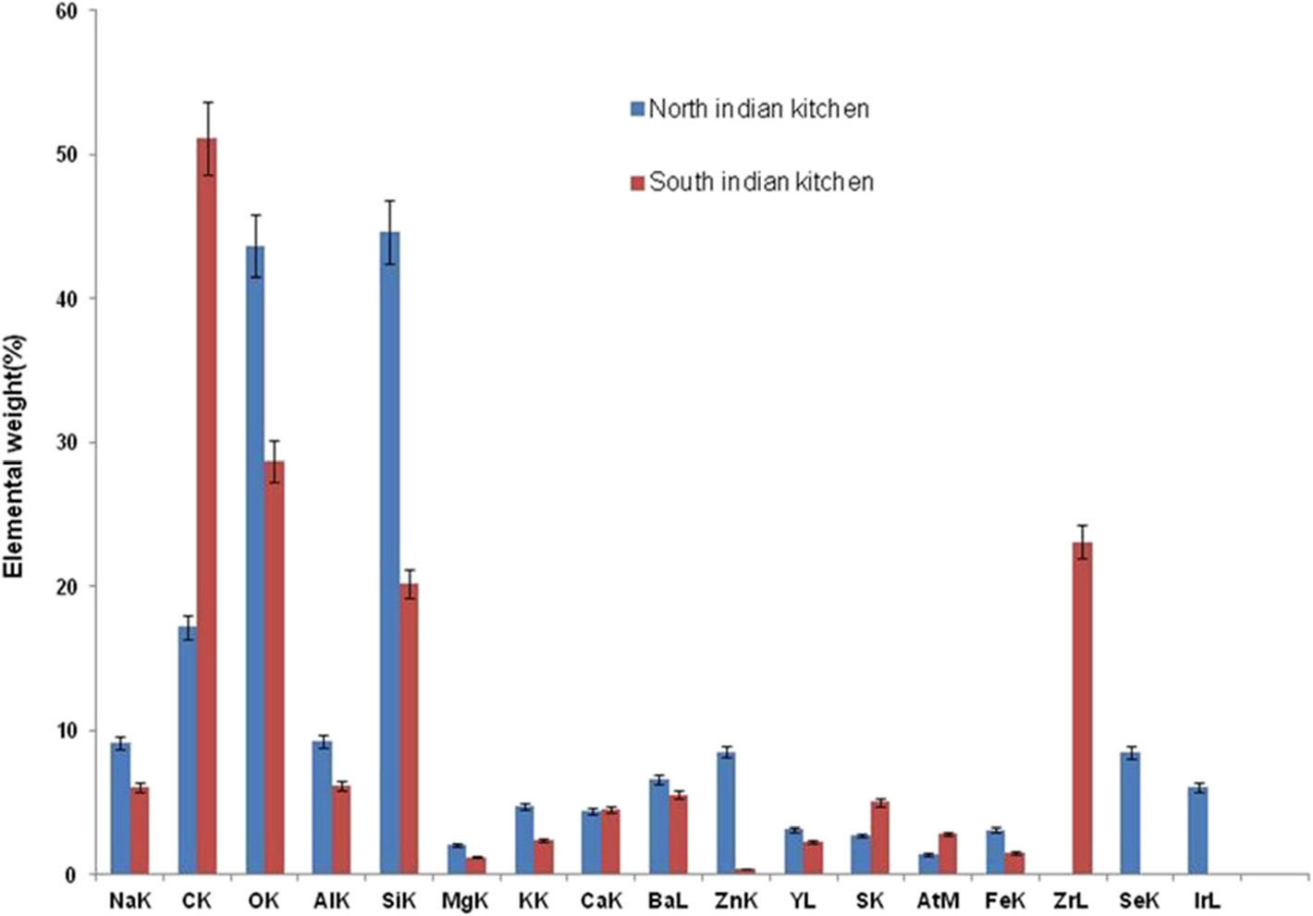
SEM-EDX analysis of kitchen indoor air sample from North India and South India

Fig. 1 shows the different shapes and sizes of indoor air particles in kitchens in north and south India. In SEM analysis of indoor air kitchen samples, varied sizes and shapes of particles [coarse PM (10–2.5 μm), fine PM (>100 nm) and UFP (<100 nm)] were detected. In north Indian kitchens, large numbers of UFP aggregated together in clusters with branches and porous shapes were observed (Fig. 1a). PM of 218.7 nm with a hard, irregular shape and a rough surface structure was also observed in kitchens (Fig. 1b). PM (1 μm size) particles with flaky outgrowths and irregular stacked structures were observed in the kitchens of south India (Fig. 1c). Similarly, coarse particles (5 μm size) with aggregated branches and tubules with a scaly appearance were also observed in the kitchens in south India (Fig. 1d). SEM-EDX analysis of indoor air filter paper samples revealed the presence of the elements Na, C, O, Al, Si, Mg, Ca, Ba, Zn, S, Fe, Zr, Se, Fe and S in the kitchens in both north and south India (Fig. 2). Higher concentrations of Na, Al, C, Zn, Si, Ca, Be, S, Mg, O, Fe and S were observed in both the kitchens (Fig. 2). The selenium concentration was exceptionally high in north Indian kitchens (Fig. 2).

**Fig. 3:**
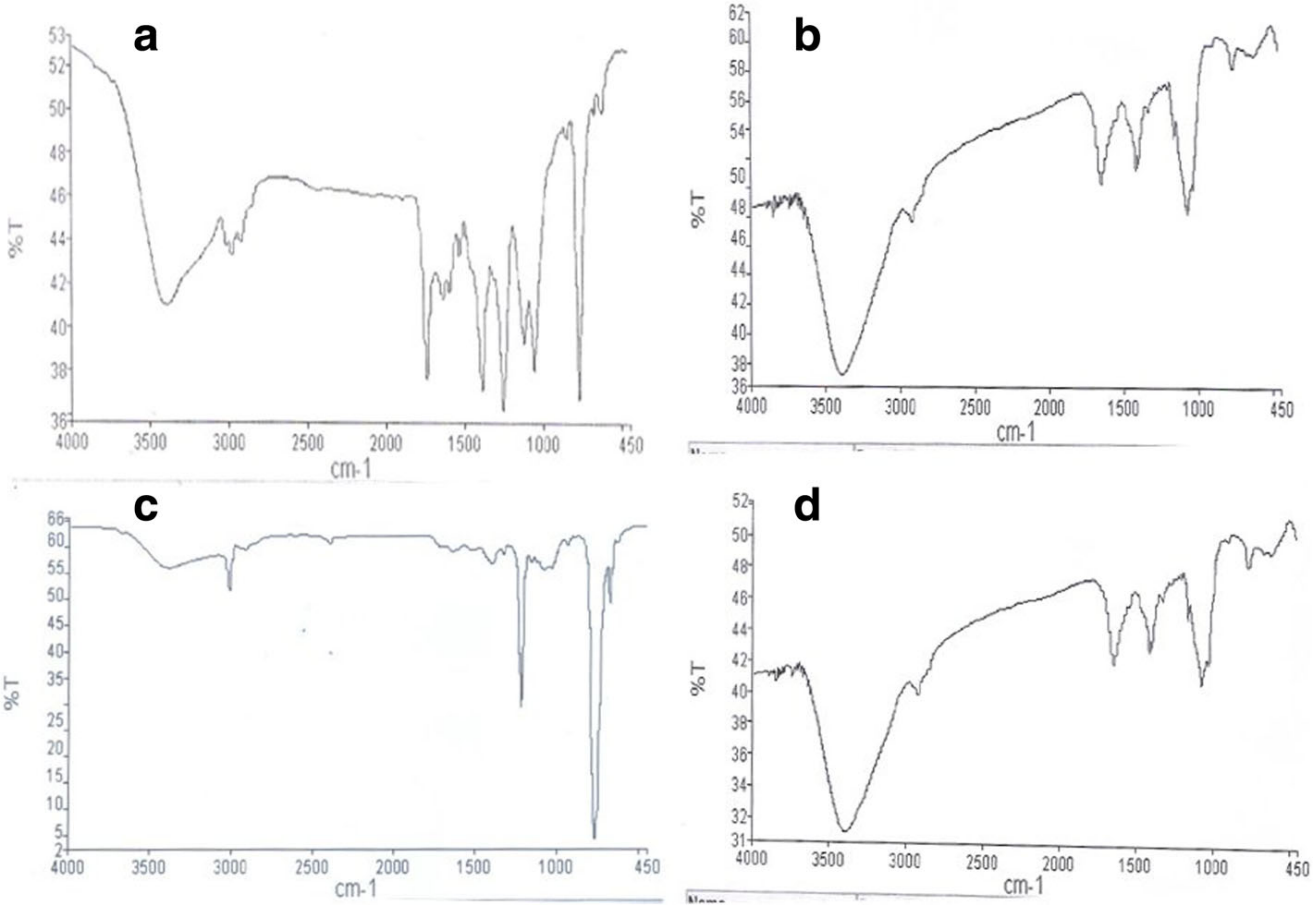
Fourier transforms infrared spectroscopy analysis of kitchen indoor air samples. FTIR spectra of different location kitchen indoor air sample :(1) North Indian kitchen (**a**,**b**) (2) South Indian Kitchen (**c**,**d**)

FTIR analysis showed the different compounds detected in the indoor air at both the kitchens (Table 5, Fig. 3). Identification of molecular structures from FTIR spectroscopy was acheived based on the absorption bands attributable to the functional groups in the respective molecules. The peaks and shoulders of the FTIR spectrum are attributed to specific functional groups on particular classes of compounds. FTIR analysis showed alkanes, alkenes, alkynes, aromatic hydrocarbons, amines, amides, alcohols, phenols, and carboxylic acids in kitchen indoor air at both locations (Table 5).

**Table 5:** Fourier transforms infrared spectroscopy analysis of kitchen indoor air samples

Functional class	Functional group	Normal range (cm^−1^)	North Indian Kitchen (Spectra detected cm^−1^)	South Indian Kitchen (Spectra detected cm^−1^)
Alkane	C-H strech	2950-2850	2925.58,2930.41	2925.99,2950,2930.36
Alkene	C-H strech	3100-3010	3020.05	3019.08,3021.27
	C = C Strech	1680-1620	ND	1634.08
Alkyne	C = C Strech	2260-2100	ND	2126.09,
Aromatic	C = C strech	1400-1600	1543.71, 1584.35	ND
Alcohol/Phenol	C-O strech	1150-1050	1068.65,1065.29,1068.25	1068.16,1080.18,1111.17
	O-H strech	3550-3200	3399.78,3402.43,3399.79	3399.88,3417.45,3393.67,3400.75,3397.31
Carboxylic Acid	O-H strech	3000-2500	ND	2520.54,2839.84
Amine	C-N strech	1080-1360	1215.3,1154.96,1266.83	1154.88,1215.71,1216.02,1218.74
Amide	C = O strech	1690-1630	1638.74	1638.84,1634.08,1644.48,1644.08

## Discussion

There is a lack of evidence on the relation among MAU, poor lung function and microvascular dysfunctions among kitchen workers in India exposed to indoor air pollutants. A major rise in nephropathy-related disorders in India can be attributed to high levels of environmental pollution [36, 37]. To the best of our knowledge, the present study is the first to investigate the poor lung function and its inverse relationship with MAU in kitchen workers after adjusting for confounding factors. The present study will serve as a pilot study for future research on this problem. Although more validation studies are required, the present study shows that albumin excretion in kitchen workers can be considered a subclinical indicator for indoor air pollution in the workplace. The cocktail of indoor air pollutants (PM_2.5_, PM_1_, UFP, TVOC, CO), elements detected in EDX and compounds detected using FTIR are apparently associated with lung function abnormalities (restrictive and obstructive), increased blood pressure and MAU in kitchen workers at both locations. Cooking oil fumes can be a source of indoor air pollutants in the kitchen, and cooking fuel has been associated with negative impacts on human health, including adverse respiratory, cardiovascular, and cardiopulmonary health outcomes [38]. Positive associations have been observed between PM_10–2.5_ and a variety of health problems including cardiovascular diseases [39], mortality [40], respiratory diseases [41] and chronic obstructive pulmonary disease (COPD) [42]. Clinical interpretation revealed that FEV_1_ reduction can be associated with increased resistance in the airways. The restrictive pattern based on FEV_6_ can be due to decreased vital capacity in the lung. The decline in PEFR can be linked to the reduced air flow rate in the bronchial airways. The increase in airway resistance can be associated with deposition of fine PM, UFP and organic compounds generated in the kitchen during cooking and combustion activities. Clinically “small” effects of air pollution on lung functions have a large public health impact as per the Swiss Study on Air Pollution and Lung Disease in Adults (SAPALDIA) [43]. Hence, these small effects observed in the present study related to the lung functions due to indoor air pollutant exposure in kitchen can have a large impact on occupational health, morbidity, loss of workdays, and future health management costs. Reduced lung function (FVC and FEV_1_) and its relationship with MAU in the general population were reported in earlier studies [20, 44, 45]. In the present study, a similar inverse relationship between lung function and MAU was observed in the kitchen workers.

The restrictive and obstructive types of lung function impairment based on FEV_6_, FEV_1_ and PEFR were strongly related to MAU in kitchen workers. MAU is considered an early indicator of kidney damage and atherosclerosis [20]. Although the pathological mechanism underlying the relationship between lung function abnormalities and blood pressure variations was not clear, previous reports have shown that lower FVC is linked to atherogenic diseases [15, 46]. Many epidemiological studies provide evidence of causal association between exposure to PM (coarse, fine and ultrafine particles) and cardiorespiratory problems [47]. Cooking can produce more than 10 times as much UFP as is produced during non-cooking periods [48]. The maximum concentration of UFP was found to be approximately 6 x 10^6^ particles per cm^3^ at 260 °C [49]. Smaller particles can cause more inflammation to the lungs than larger particles of a similar elemental composition [50].

In addition to technical factors related to equipment and procedures, biological and environmental factors are other sources of variation in lung functions. These include race, ethnicity, sex, environmental influences, nutrition, childhood infections, and other undefined factors [51]. Since the kitchen workers are male and have similar anthropometric parameters, ethnic differences in lung functions may be another reason for higher lung function in north Indian workers. The higher lung function in the north Indian population than in their south Indian counterparts was evidenced in earlier studies [51, 52]. Lung function impairments such as obstruction and restriction also show higher risk among south Indian kitchen workers than that among their north Indian counterparts. This risk difference can also be attributed to the ethnic difference of lung functions, apart from their exposure to indoor air pollutants.

In the present study, SEM-EDX analysis provides useful information on the morphology and elemental composition of indoor air pollutants. SEM-EDX analysis provides insights into the origin of particles such as whether they are emitted from anthropogenic or natural processes [53]. Kitchen indoor air contains fine particles and UFP [49, 54]. The particles contain a diverse range of elements, which may have adverse effects on airflow rates and lung volumes along with kidney dysfunctions such as microalbuminuria. Transition metals in PM were considered causative factors for several adverse health effects [55]. Iron particles generated during combustion processes [56] that are more soluble and bio-available than iron in mineral form can have higher health implications [57].

Carbon and oxygen in the particles can be generated from burning organic or inorganic oxides, acids and/or salts present in food or oil [58]. Ca, K, Fe, Si and Al can be generated from indoor or outdoor air during burning of crustal material and combustion of fossil and biomass fuels. The sources of these elements in kitchen can be cooking gas (LPG), utensils or minerals present in food. The human body requires a small amount of essential elements or trace elements for cell function at the biological, chemical and molecular levels [59], and vegetables and other foods are sources of these elements. Therefore, these elements in kitchen indoor air probably come from food sources or cross-boundary movement of particles from the ambient air. Since the oils used in these mega-kitchens are refined vegetable oils, the type of food fried and cooked, cooking fuel leakage and limitations in ventilation in the kitchens may be the reasons for the high TVOC and CO_2_ in the kitchens in north India compared to those in south India.

Fine particle and UFP deposition within the airways during inhalation is capable of increasing airway resistance, causing lung function deficits, autonomic nervous system imbalance and sometimes increases in blood pressure, as reported in an earlier study [60]. Certain particle constituents (e.g., metals, organic compounds) might also be capable of reaching the systemic circulation upon inhalation and thereafter directly impair microvascular function, leading to MAU [60]. The risk of cardiovascular morbidity and mortality increases with the severity of obstructive and restrictive lung function patterns [31]. Similar patterns of obstructive and restrictive lung function cases were observed in the kitchen workers.

Fat aerosols contain a mixture of heat- and water-treated fat from fried meat, hydrolyzed vegetable fat and other degradation products such as fatty acids, other organic acids and aldehydes [61, 62]. Incomplete combustion of organic materials produces a complex mixture of chemicals, many of which are known to be irritants, toxic or carcinogenic to humans [62]. FTIR spectral analysis of kitchen indoor air showed different types of functional groups that may be possible causative agents for reduced lung function and enhanced risk of microalbuminuria among kitchen workers. Cooking can generate appreciable masses of aerosols within the area where cooking occurs. Earlier studies have also shown these particle to be largely within the respirable size ranges, containing organic chemical groups such as alkanes, fatty acids, and alkanones [63].

The organic and inorganic compounds associated with particle fractions (PM_2.5_, PM_1_) in kitchen air might accumulate in the tracheobronchial epithelium, where they could increase in concentration even at low environmental exposure. The gas phase compounds or those that rapidly elute from particles in the kitchen upon inhalation may reach the alveolar epithelium and rapidly enter the circulatory system in kitchen workers. A linear trend between level of exposure to PM and measures of poor kidney function (albumin-to-creatinine ratio – ACR) was also observed among residents living adjacent to the Twin Towers in the USA [29]. This report [29] supports our observation of higher PM concentration in indoor air and its etiological association with poor ACR.

The possible association between MAU and cardiovascular disease can consist of either MAU causing cardiovascular disease or cardiovascular disease causing MAU. Both conditions could also be simultaneously caused by a common risk factor or pathophysiologic process [64]. MAU is a primary independent surrogate marker of kidney damage and cardiovascular problems [65, 66]. Higher levels of albumin in the urine result from physiological alterations such as blood pressure variations in the glomerulus and changes in the glomerular capillary basement membrane, possibly due to glycosylation of proteins in the membrane [67]. An increased level of albuminuria is an indicator of blood albumin leakage due to systemic inflammation and endothelial dysfunction [68]. Poor lung function can be associated with vascular damage and endothelial dysfunction through systemic inflammation [69, 70]. Although the pathological mechanism is not clearly understood, deposition of inhaled PM can alter upper and lower airway status and causes systemic inflammation and endothelial damage [71, 72] that leads to blood albumin leakage from tubular endothelial damage [73].

The mechanism by which long term-exposure to fine particulate air pollution may increase the risk of kidney dysfunction or membranous nephropathy remains to be explained [36]. Membranous nephropathy is considered an autoimmune disease characterized by the formation of circulating autoantibodies and subepithelial immune complex deposits in the kidney [36, 74]. In vivo studies have observed that exposure to fine PM promotes the production of autoantibodies and immune complexes [75, 76]. It has been hypothesized that cytokines generated in the airways in response to air pollution can spill over into the circulation, which further influences the autoimmune responses [77]. Air pollution also increases the circulating levels of inflammation mediators such as TNF-a, IL-6, and plasminogen activator inhibitor [78, 79].

MAU may be a causative factor for nephropathy in patients with diabetes [26]. Some studies reported that MAU is the most common cause of mortality in hypertensive subjects with or without type 2 diabetes mellitus (DM) [28] and in non-hypertensive subjects [80]. A Korean population study revealed a strong relationship between poor lung function and MAU or UACR after adjusting for confounding factors such as age, hypertension or SBP, diabetes mellitus, triglyceride level, obesity and smoking [20]. Although we adjusted our data for confounding factors, the role of indoor air pollutants in such association is still not clear. This is mainly because of the synergistic interaction of multiple kitchen indoor air pollutants including different fractions of PM on the physiological mechanisms of the respiratory and excretory systems. More in vivo and in vitro studies based on inhalation toxicology experiments are required to substantiate our findings further.

The current study has limitation and strengths. Due to the cross-sectional study design, the direction of causality in our study is not very evident. Owing to limitations of time and resources, a follow-up study could not be undertaken, which can be considered a limitation of the study. A follow-up study could have given a more precise measure of the risk associated with poor lung function and microalbuminuria. No proper database is available on kitchen workers, as this profession is an unorganized or informal sector in India. The sufficient sample size in both the MAU and non-MAU groups and the inclusion of two regional study locations in India are the main strengths of the study. Based on the study findings, it is suggested that regulatory authorities in India should propose new national indoor air quality standards and policies for commercial kitchens.

## Conclusions

In conclusion, this study is the first report on poor lung function and its association with MAU among kitchen workers in India exposed to multiple indoor air pollutants. Kitchen workers in south India showed lower lung capacities and a greater risk of obstructive and restrictive abnormalities than their north Indian counterparts. This can be attributed to their ethnicity, apart from their workplace exposure to indoor air pollutants. The deposition of fine and ultrafine particles and other indoor air contaminants can not only alter the lung function but also affect microvascular functions, viz*.*, microalbuminuria through the possible route of systemic circulation. In the future, more experimental and occupational health studies should be conducted on this problem to further validate the findings of the present study.

## Abbreviations

ACR: Albumin -to-creatinine ratio
BMI: Body mass index
BP: Blood pressure
CO: Carbon monoxide
CO_2_: Carbon dioxide
DBP: Diastolic Blood Pressure
FEV: Forced expiratory volume
FTIR: Fourier transform infra red spectroscopy
FVC: Forced vital capacity
IDF: International Diabetes Federation
IQM: Indoor air quality monitor
MAU: Microalbuminuria
non-MAU: Without microalbuminuria
PAH: Polyaromatic hydrocarbon
PEF: Peak expiratory flow rate
PM: Particulate matter
SBP: Systolic Blood Pressure
SEM: Scanning electron microscopy
TVOC: Total volatile organic compounds
UFP: Ultrafine particles
WHO: World health organisation

## References

[1] Singh A, Chandrasekharan Nair K, Kamal R, Bihari V, Gupta MK, Mudiam MK, Satyanarayana GN, Raj A, Haq I, Shukla NK (2016). Assessing hazardous risks of indoor airborne polycyclic aromatic hydrocarbons in the kitchen and its association with lung functions and urinary PAH metabolites in kitchen workers. Clin Chim Acta.

[2] Sjaastad AK, Jorgensen RB, Svendsen K (2010). Exposure to polycyclic aromatic hydrocarbons (PAHs), mutagenic aldehydes and particulate matter during pan frying of beefsteak. Occup Environ Med.

[3] Uzoigwe JC, Prum T, Bresnahan E, Garelnabi M (2013). The emerging role of outdoor and indoor air pollution in cardiovascular disease. N Am J Med Sci.

[4] Pope CA, Burnett RT, Thurston GD, Thun MJ, Calle EE, Krewski D, Godleski JJ (2004). Cardiovascular mortality and long-term exposure to particulate air pollution: epidemiological evidence of general pathophysiological pathways of disease. Circulation.

[5] WHO. 7 million premature deaths annually linked to air pollution. 2014. http://www.who.int/mediacentre/news/releases/2014/air-pollution/en/. Accessed 18 Aug 2016.

[6] Harris G. Beijing’s Air would Be called good in Delhi. In: The New York Times international weekly. 2014. https://www.nytimes.com/2014/01/26/world/asia/beijings-air-would-be-step-up-for-smoggy-delhi.html?_r=0. Accessed 22 Aug 2016.

[7] Sjaastad AK, Svendsen K (2008). Exposure to mutagenic aldehydes and particulate matter during panfrying of beefsteak with margarine, rapeseed oil, olive oil or soybean oil. Ann Occup Hyg.

[8] Daigle CC, Chalupa DC, Gibb FR, Morrow PE, Oberdorster G, Utell MJ, Frampton MW (2003). Ultrafine particle deposition in humans during rest and exercise. Inhal Toxicol.

[9] Chin MT (2015). Basic mechanisms for adverse cardiovascular events associated with air pollution. Heart.

[10] Oberdorster G, Oberdorster E, Oberdorster J (2005). Nanotoxicology: an emerging discipline evolving from studies of ultrafine particles. Environ Health Perspect.

[11] Shah AS, Langrish JP, Nair H, McAllister DA, Hunter AL, Donaldson K, Newby DE, Mills NL (2013). Global association of air pollution and heart failure: a systematic review and meta-analysis. Lancet.

[12] Stafoggia M, Cesaroni G, Peters A, Andersen ZJ, Badaloni C, Beelen R, Caracciolo B, Cyrys J, de Faire U, de Hoogh K (2014). Long-term exposure to ambient air pollution and incidence of cerebrovascular events: results from 11 European cohorts within the ESCAPE project. Environ Health Perspect.

[13] Bellavia A, Urch B, Speck M, Brook RD, Scott JA, Albetti B, Behbod B, North M, Valeri L, Bertazzi PA (2013). DNA hypomethylation, ambient particulate matter, and increased blood pressure: findings from controlled human exposure experiments. J Am Heart Assoc.

[14] Vestbo J, Hurd SS, Agustí AG, Jones PW, Vogelmeier C (2013). Global strategy for the diagnosis, management, and prevention of chronic obstructive pulmonary disease : GOLD executive summary. Am J Respir Crit Care Med.

[15] Johnston AK, Mannino DM, Hagan GW, Davis KJ, Kiri VA (2008). Relationship between lung function impairment and incidence or recurrence of cardiovascular events in a middle-aged cohort. Thorax.

[16] Nemmar A, Hoet PH, Vanquickenborne B, Dinsdale D, Thomeer M, Hoylaerts MF, Vanbilloen H, Mortelmans L, Nemery B (2002). Passage of inhaled particles into the blood circulation in humans. Circulation.

[17] Brook RD, Rajagopalan S, Pope CA, Brook JR, Bhatnagar A, Diez-Roux AV, Holguin F, Hong Y, Luepker RV, Mittleman MA (2010). Particulate matter air pollution and cardiovascular disease: An update to the scientific statement from the American Heart Association. Circulation.

[18] Zhang YH, Gao Y, Mao X, Shang J, Su BL. Assessment of carotid atherosclerosis in type 2 diabetes mellitus patients with microalbuminuria by high-frequency ultrasonography. Int J Endocrinol. 2013. doi:10.1155/2013/819584 10.1155/2013/819584 PMC3612477 23573090

[19] Mannino DM, Doherty DE, Sonia BA (2006). Global Initiative on Obstructive Lung Disease (GOLD) classification of lung disease and mortality: findings from the Atherosclerosis Risk in Communities (ARIC) study. Respir Med.

[20] Yoon JH, Won JU, Ahn YS, Roh J (2014). Poor lung function has inverse relationship with microalbuminuria, an early surrogate marker of kidney damage and atherosclerosis: the 5th Korea National Health and Nutrition Examination Survey. PLoS One.

[21] Thoenes M, Bramlage P, Khan BV, Schieffer B, Kirch W, Weir MR (2007). Albuminuria: pathophysiology, epidemiology and clinical relevance of an emerging marker for cardiovascular disease. Future Cardiol.

[22] ADA (2001). Clinical practice recommendations 2001: diabetic nephropathy. Diabetes Care.

[23] Chen B, Yang D, Chen Y, Xu W, Ye B, Ni Z (2010). The prevalence of microalbuminuria and its relationships with the components of metabolic syndrome in the general population of China. Clin Chim Acta.

[24] Ozyilmaz A, Bakker SJ, de Zeeuw D, de Jong PE, Gansevoort RT (2010). Selection on albuminuria enhances the efficacy of screening for cardiovascular risk factors. Nephrol Dial Transplant.

[25] Gerstein HC, Mann JF, Yi Q, Zinman B, Dinneen SF, Hoogwerf B, Halle JP, Young J, Rashkow A, Joyce C (2001). Albuminuria and risk of cardiovascular events, death, and heart failure in diabetic and nondiabetic individuals. JAMA.

[26] Karalliedde J, Viberti G (2004). Microalbuminuria and cardiovascular risk. Am J Hypertens.

[27] Furtner M, Kiechl S, Mair A, Seppi K, Weger S, Oberhollenzer F, Poewe W, Willeit J (2005). Urinary albumin excretion is independently associated with carotid and femoral artery atherosclerosis in the general population. Eur Heart J.

[28] Jorgensen L, Jenssen T, Johnsen SH, Mathiesen EB, Heuch I, Joakimsen O, Fosse E, Jacobsen BK (2007). Albuminuria as risk factor for initiation and progression of carotid atherosclerosis in non-diabetic persons: the Tromso Study. Eur Heart J.

[29] McLaughlin M. 9/11 responders suffered kidney damage due to air pollutants. American Society of Nephrology. 2013. https://www.eurekalert.org/pub_releases/2013-11/ason-9rs102113.php. Accessed 15 Sept 2016.

[30] Miller MR, Hankinson J, Brusasco V, Burgos F, Casaburi R, Coates A, Crapo R, Enright P, van der Grinten CP, Gustafsson P (2005). Standardisation of spirometry. Eur Respir J.

[31] Gruffydd-Jones K, Loveridge C (2011). The 2010 NICE COPD Guidelines: how do they compare with the GOLD guidelines?. Prim Care Respir J.

[32] Udwadia FE, Sunavala JD, Shetye VM, Jain PK (1986). The maximal expiratory flow-volume curve in normal subjects in India. Chest.

[33] Farmer AJ, Stevens R, Hirst J, Lung T, Oke J, Clarke P, Glasziou P, Neil A, Dunger D (2014). Optimal strategies for identifying kidney disease in diabetes: properties of screening tests, progression of renal dysfunction and impact of treatment - systematic review and modelling of progression and cost-effectiveness. Health Technol Assess.

[34] Zhu L, Wang J (2003). Sources and patterns of polycyclic aromatic hydrocarbons pollution in kitchen air, China. Chemosphere.

[35] Meng QY, Turpin BJ, Korn L, Weisel CP, Morandi M, Colome S, Zhang JJ, Stock T, Spektor D, Winer A (2005). Influence of ambient (outdoor) sources on residential indoor and personal PM2.5 concentrations: analyses of RIOPA data. J Expo Anal Environ Epidemiol.

[36] Xu X, Wang G, Chen N, Lu T, Nie S, Xu G, Zhang P, Luo Y, Wang Y, Wang X, et al. Long-Term Exposure to Air Pollution and Increased Risk of Membranous Nephropathy in China. J Am Soc Nephrol. 2016;27(12):3739–746. 10.1681/ASN.2016010093 PMC5118492 27365535

[37] Narasimhan B, Chacko B, John GT, Korula A, Kirubakaran MG, Jacob CK (2006). Characterization of kidney lesions in Indian adults: towards a renal biopsy registry. J Nephrol.

[38] Pope CA. Mortality effects of longer term exposures to fine particulate air pollution: review of recent epidemiological evidence. Inhal Toxicol. 2007;19 Suppl 1:33–8. doi:10.1080/08958370701492961. 10.1080/08958370701492961 17886048

[39] Chang HH, Peng RD, Dominici F (2011). Estimating the acute health effects of coarse particulate matter accounting for exposure measurement error. Biostatistics.

[40] Malig BJ, Ostro BD (2009). Coarse particles and mortality: evidence from a multi-city study in California. Occup Environ Med.

[41] Host S, Larrieu S, Pascal L, Blanchard M, Declercq C, Fabre P, Jusot JF, Chardon B, Le Tertre A, Wagner V (2008). Short-term associations between fine and coarse particles and hospital admissions for cardiorespiratory diseases in six French cities. Occup Environ Med.

[42] Brunekreef B, Forsberg B (2005). Epidemiological evidence of effects of coarse airborne particles on health. Eur Respir J.

[43] Kunzli N, Ackermann-Liebrich U, Brandli O, Tschopp JM, Schindler C, Leuenberger P (2000). Clinically “small” effects of air pollution on FVC have a large public health impact. Swiss Study on Air Pollution and Lung Disease in Adults (SAPALDIA)-team. Eur Respir J.

[44] Kataria A, Trasande L, Trachtman H (2015). The effects of environmental chemicals on renal function. Nat Rev Nephrol.

[45] Takase H, Sugiura T, Ohte N, Dohi Y (2015). Urinary albumin as a marker of future blood pressure and hypertension in the general population. Medicine.

[46] Yoon JH, Choi BS, Shin JH (2012). Association between impaired lung function and coronary artery calcium score in workers exposed to inorganic dust. Toxicol Environ Heal Sci.

[47] Pope CA (2000). Epidemiology of fine particulate air pollution and human health: biologic mechanisms and who’s at risk?. Environ Health Perspect.

[48] Wallace LA, Emmerich SJ, Howard-Reed C (2004). Source strengths of ultrafine and fine particles due to cooking with a gas stove. Environ Sci Technol.

[49] Tseng LC, Chen CC (2013). Effect of flow characteristics on ultrafine particle emissions from range hoods. Ann Occup Hyg.

[50] Srivastava A, Jain VK, Srivastava A (2009). SEM-EDX analysis of various sizes aerosols in Delhi India. Environ Monit Assess.

[51] Aggarwal AN, Gupta D, Jindal SK (2007). Comparison of Indain reference equations for spirometry interpretation. Respirology.

[52] Chhabra SK (2009). Regional variations in vital capacity in adult males in India: comparison of regression equations from four regions and impact on interpretation of spirometric data. Ind J Chest Dis Allied Sci.

[53] Cong Z, Kang S, Dong S, Liu X, Qin D (2010). Elemental and individual particle analysis of atmospheric aerosols from high Himalayas. Environ Monit Assess.

[54] Torkmahalleh MA, Goldasteh I, Zhao Y, Udochu NM, Rossner A, Hopke PK, Ferro AR (2012). PM2.5 and ultrafine particles emitted during heating of commercial cooking oils. Indoor Air.

[55] Chen LC, Lippmann M (2009). Effects of metals within ambient air particulate matter (PM) on human health. Inhal Toxicol.

[56] Deguillaume L, Leriche M, Desboeufs K, Mailhot G, George C, Chaumerliac N (2005). Transition metals in atmospheric liquid phases: sources, reactivity, and sensitive parameters. Chem Rev.

[57] Luo C, Mahowald N, Bond T, Chuang PY, Artaxo P, Siefert R, Chen Y, Schauer J. Combustion iron distribution and deposition. Global Biogeochemical Cycles. 2008. doi:10.1029/2007GB002964.

[58] Estokova A, Stevulova N, Kubincová L (2010). Particulate matter investigation in indoor air environment. Global Nest J.

[59] Prashanth L, Kattapagari K, Chitturi R, Baddam V, Prasad L (2015). A review on role of essential trace elements in health and disease. J Dr NTR Univ Health Sci.

[60] Brook RD, Rajagopalan S (2009). Particulate matter, air pollution, and blood pressure. J Am Soc Hypertens.

[61] Svendsen K, Jensen HN, Sivertsen I, Sjaastad AK (2002). Exposure to cooking fumes in restaurant kitchens in norway. Ann Occup Hyg.

[62] Wang T (2007). Particulate matter from Chinese cooking. Environ Sci Technol.

[63] Karimatu LA, Juana MDS, Roy MH (2013). Emissions and indoor concentrations of particulate matter and its specific chemical components from cooking: A review. Atmos Environ.

[64] Stehouwer CD, Smulders YM (2006). Microalbuminuria and risk for cardiovascular disease: Analysis of potential mechanisms. J Am Soc Nephrol.

[65] Roscioni SS, Lambers Heerspink HJ, de Zeeuw D (2014). Microalbuminuria: target for renoprotective therapy PRO. Kidney Int.

[66] Currie G, Delles C (2013). Proteinuria and its relation to cardiovascular disease. Int J Nephrol Renovasc Dis.

[67] Goetz FC, Jacobs DR, Chavers B, Roel J, Yelle M, Sprafka JM (1997). Risk factors for kidney damage in the adult population of Wadena, Minnesota. A prospective study. Am J Epidemiol.

[68] Jacobsen LM, Winsvold BS, Romundstad S, Pripp AH, Holmen J, Zwart JA. Urinary albumin excretion as a marker of endothelial dysfunction in migraine sufferers: The HUNT study, Norway. BMJ Open. 2013;3:e003268. doi:10.1136/bmjopen-2013-003268. 10.1136/bmjopen-2013-003268 PMC3740253 23943777

[69] van Gestel YR, Flu WJ, van Kuijk JP, Hoeks SE, Bax JJ, Sin DD, Poldermans D (2010). Association of COPD with carotid wall intima-media thickness in vascular surgery patients. Respir Med.

[70] Akpinar EE, Akpinar S, Ertek S, Sayin E, Gulhan M (2012). Systemic inflammation and metabolic syndrome in stable COPD patients. Tuberk Toraks.

[71] von Bornstadt D, Kunz A, Endres M (2014). Impact of particulate matter exposition on the risk of ischemic stroke: epidemiologic evidence and putative mechanisms. J Cereb Blood Flow Metab.

[72] Li Y, Rittenhouse-Olson K, Scheider WL, Mu L (2012). Effect of particulate matter air pollution on C-reactive protein: a review of epidemiologic studies. Rev Environ Health.

[73] Koroshi A (2007). Microalbuminuria, is it so important?. Hippokratia.

[74] Ponticelli C, Glassock RJ (2014). Glomerular diseases: membranous nephropathy—a modern view. Clin J Am Soc Nephrol.

[75] Pfau JC, Brown JM, Holian A (2004). Silica-exposed mice generate autoantibodies to apoptotic cells. Toxicology.

[76] Brown JM, Pfau JC, Holian A (2004). Immunoglobulin and lymphocyte responses following silica exposure in New Zealand mixed mice. Inhal Toxicol.

[77] Ritz SA (2010). Air pollution as a potential contributor to the ‘epidemic’ of autoimmune disease. Med Hypotheses.

[78] Thompson AM, Zanobetti A, Silverman F, Schwartz J, Coull B, Urch B, Speck M, Brook JR, Manno M, Gold DR (2010). Baseline repeated measures from controlled human exposure studies: associations between ambient air pollution exposure and the systemic inflammatory biomarkers IL-6 and fibrinogen. Environ Health Perspect.

[79] Panasevich S, Leander K, Rosenlund M, Ljungman P, Bellander T, de Faire U, Pershagen G, Nyberg F (2009). Associations of long- and short-term air pollution exposure with markers of inflammation and coagulation in a population sample. Occup Environ Med.

[80] Hillege HL, Janssen WMT, Bak AAA, Diercks GFH, Grobbee DE (2001). Microalbuminuria is common, also in a nondiabetic, nonhypertensive population, and an independent indicator of cardiovascular risk factors and cardiovascular morbidity. J Intern Med.

